# Social and behavioral interventions for improving quality of life of HIV infected people receiving antiretroviral therapy: a systematic review and meta-analysis

**DOI:** 10.1186/s12955-017-0662-4

**Published:** 2017-04-24

**Authors:** Dharma Nand Bhatta, Tippawan Liabsuetrakul, Edward B. McNeil

**Affiliations:** 1 Department of Community Medicine and Public Health, Tribhuvan University, Peoples’ Dental College, Kathmandu, Nepal; 2 Faculty of Medicine, Epidemiology Unit, Prince of Songkla University, Hat Yai, Thailand; 3 Department of Public Health, Pokhara University, Nobel College, Kathmandu, Nepal

**Keywords:** Social intervention, Behavioural intervention, Randomized, HIV, Quality of life, ART

## Abstract

**Background:**

Improvement in quality of life is crucial for HIV infected people. Social and behavioral interventions have been implemented in different contexts to improve the quality of life among HIV infected people. This review appraises the evidence for available interventions that focused on quality of life of HIV infected people receiving antiretroviral therapy (ART).

**Methods:**

We searched electronic databases for randomized controlled trials of interventions to improve the quality of life of HIV infected people receiving ART. We searched PUBMED and the Cochrane Centre Register of Controlled Trials (CENTRAL) with the terms “social”, “behavioral”, “educational”, “quality of life”, “HIV”, and “RCT”. Searches were conducted for articles published from 1980 to December 16, 2015. Standardized data abstraction methods and searching steps were applied.

**Results:**

Twenty-eight studies reported the impact of social or behavioral interventions in quality of life among HIV infected people, of which 15 were conducted in United States of America. A total of 4136 participants were enrolled. Of the 28 studies, four studies included females, two studies included males and remaining studies excluded both males and females. The overall reported methodological quality of the studies was subject to a high risk of bias and the study criteria were unclear in most studies. Twenty-one studies reported a significant intervention effect on at least one quality of life domain. Meta-analyses showed significant improvement in general health, mental health, physical function and environment domains of quality of life among intervention groups. However, the expected impact of the intervention was low to moderate because the rigorousness of the studies was low, information was limited, the sample sizes were small and other the quality of the study designs were poor.

**Conclusions:**

Although the available evidence suggests that existing social and behavioral interventions can improve some quality of life domains, the quality of evidence was insufficient to support the notion that these interventions can improve the overall quality of life of HIV infected people receiving ART. Well-designed and rigorous randomized controlled trials with high methodological quality are required.

## Background

Globally, at the end of 2015, there were approximately 16 million people receiving antiretroviral therapy (ART) out of 36.9 million HIV infected people [1]. United Nation’s agencies developed the 90-90-90 strategy (90% of all people living with HIV will know their HIV status, 90% of all people with diagnosed HIV infection will receive sustained ART and 90% of all people receiving ART will have viral suppression) to be achieved by the year 2020 that will help end HIV epidemics [2]. Revised comprehensive ART guidelines developed by World Health Organization (WHO) [3] aim to help prevent the HIV burden and improve the quality of life of HIV infected people. ART has several public health benefits [4, 5] but its side effects can alter adherence and affect quality of life [6]. Environmental, social, structural, and personal factors can also affect adherence to ART and quality of life [7].

The concept of quality of life is: “an individual’s perception of their position in life in the context of the culture and value systems in which they live and in relation to their goals, expectations, standards and concerns” [8]. Quality of life may be improved through different coping mechanisms, self-efficacy, social, psychological, structural and environmental adjustments. HIV burden is associated with clinical, psychological, behavioral and social problems. Various studies measured the quality of life which covers psychological, social and behavioral aspect [9–11]. Intervention with revised component into the routine health care services may help to improve the quality of life of HIV infected people.

Behavioral or social interventions are developed based on different theories that changes an individual’s psychological and social attributes [12]. Behavioral or social interventions provide the chance to share experiences among groups which would help to reduce the loneliness, negative feelings and stress of HIV infected people [13]. Motivation, social and psychological support enhances empowerment and social support among HIV infected populations to access health care services, improve their quality of life and ability to cope with stigma [14, 15]. Furthermore, involvement in the intervention can improve the rate of retention in care or adherence to ART, reduce risky sexual behaviors and increase negotiation skills, self-efficacy, social networks, and communication skills [10, 16]. Systematically integrated behavioral or social interventions for HIV infected people may help to improve access to health care service, reduce the risk of transmission and improve quality of life.

Several interventions have been developed around the world to improve the quality of life of HIV infected people. Comprehensive systematic reviews and meta-analyses based on interventions that focus on improving quality of life among HIV infected individuals receiving ART are lacking and existing evidence on the effectiveness of preventive interventions is limited [17]. Systematic reviews of studies found that interventions which covered support groups, social services and combined aerobic and resistance exercises had a low to moderate impact on the quality of life of HIV infected people [18–20]. Evidence based findings are required to prioritize activities and resources and develop strategically planned policies. The aim of this review is to summarize the available evidence for behavioral and social interventions for HIV infected populations in order to provide a direction to funding agencies, policy makers, planners and program developers on how best to use their resources to improve the quality of life of HIV infected people.

## Methods

### Search methods for identification of studies

We searched the literature in MEDLINE/PUBMED and Cochrane Library databases from 1980 to 16 December 2015. We developed a standard protocol for the literature search and used standard Mesh terms for PUBMED and Cochrane Centre Register of Controlled Trials (CENTRAL).

The search strategy focused on three keywords: study participants, design and interventions. The search strategy was as follows:

1. social OR behavioral OR behavioural OR educational
2. quality of life OR QoL
3. HIV OR human immunodeficiency virus
4. drug OR medication OR clinical
5. RCT OR randomized

- #1 AND #2 AND #3 NOT #4 AND #5

All the studies were searched by two independent authors. Search process and strategy, search record and retrieved studies were reported and documented as per the PRISMA guidelines. Duplicate citations were checked and removed by importing search results to a reference management software system. Only publications in peer-reviewed journals and in English were considered.

### Eligibility criteria

This review included all the randomized controlled trials conducted among HIV infected populations who were aged more than or equal 18 years and receiving ART. Social, behavioral or educational interventions were compared with control groups. The quality of life of HIV infected people receiving ART was reported to be one of outcome measures in the studies. The outcomes must have been compared at baseline and a predefined follow up time period.

### Data collection and analysis

Two authors independently screened the studies returned using the predefined keyword search strategy. Relevant study titles and abstracts were independently evaluated. The full text article was obtained for complete assessment after authors considered eligibility based on the title and abstract. Full text of the articles was reviewed by two reviewers independently to assess the inclusion criteria. Final selection of the articles was made by agreement between the two reviewers.

Information abstracted from each study included authors, year published, country, study design, settings, sample size, characteristics of participants, theory used, eligibility criteria, comparison group intervention, intervention components, intervention methods and duration, measurement tools and outcomes. Retention rate in the intervention group, missing data and follow up period were also extracted. The data, independently extracted by two reviewers, were compared and a consensus was made after discussion of discrepancies. The study authors were contacted to obtain clarification of missing or insufficient data.

### Assessment of risk of bias in included studies

Cochrane Handbook for Systematic Reviews of Interventions [21] guidelines were used to assess the risk of bias in all studies included in the review. Assessment criteria included random sequence generation, allocation concealment, incomplete outcomes reporting of sources of bias, blinding of participants, researcher or outcome assessors, completeness of outcome data, selectivity of outcome reporting, missing data and retention rate. The two review authors rated the risk of bias by assessing either “yes” (low risk of bias), “no” (high risk of bias) or “unclear” (insufficient information) to all probable sources of bias. Criteria for rating internal and external validity (good, fair, poor), quality of evidence for each individual study (Table 1, rated as strong, medium, weak), overall quality of the body of evidence by outcome of interest (Table 2, rated as good, fair, poor) and expected impact of the intervention on the outcome of interest (Table 3, rated as high, moderate, low, uncertain) were adapted from the United States Preventive Services Task Force procedure manual [22, 23].

**Table 1:** Criteria for rating the quality of evidence for individual studies

Level of Evidence	Description
1 = Strong	Systematic review/meta-analysis of RCTs with consistent findings; high-quality individual RCT
2 = Medium	Systematic review/meta-analysis of lower-quality clinical trials or of studies with inconsistent findings; lower quality clinical trial; cohort study; case–control study
3 = Weak	Consensus guidelines; usual practice; expert opinion; case series

**Table 2:** Criteria for rating the overall quality of the body of evidence by outcome of interest

Rating	Description
1 = Good	Evidence includes consistent results from well-designed well-conducted studies in representative populations that directly assess effects on health outcomes
2 = Fair	Evidence is sufficient to determine effects on health outcomes, but the strength of the evidence is limited by the number, quality, or consistency of the individual studies, generalizability to routine practice, or indirect nature of the evidence on health outcomes
3 = Poor	Evidence is based on consensus, usual practice, opinion, or case series. Additionally evidence is insufficient to fully assess the effects on health outcomes because of limited number, or power of studies, important flaws in design or conduct, gaps in the chain of evidence, or lack of information on importance on the key health outcomes

**Table 3:** Criteria for rating the expected impact of the intervention on the outcome of interest

Grade	Definition
High	We are very confident that the estimate of effect lies close to the true effect for this outcome. The body of evidence has few or no deficiencies. We believe that the findings are stable (i.e., another study would not change the conclusions).
Moderate	We are moderately confident that the estimate of effect lies close to the true effect for this outcome. The body of evidence has some deficiencies. We believe that the findings are likely to be stable, but some doubt remains.
Low	We have limited confidence that the estimate of effect lies close to the true effect for this outcome. The body of evidence has major or numerous deficiencies (or both). We believe that additional evidence is needed before concluding either that the findings are stable or that the estimate of effect is close to the true effect.
Insufficient	We have no evidence, we are unable to estimate an effect, or we have no confidence in the estimate of effect for this outcome. No evidence is available or the body of evidence has unacceptable deficiencies, precluding us from reaching a conclusion.

### Statistical assessment

Pooled effects were obtained by calculating standardized mean differences with 95% confidence intervals. Analysis was done using both fixed-effects and random-effects models. When there was more than one intervention arm, the mean and standard deviation was combined using Review Manager Calculator. We compared the social and behavioral intervention versus a control group. Statistical heterogeneity of the reviewed studies was assessed using Cochran's Q-test, quantified using I^2^ and categorized as low (0–25%), moderate (26–50%) or high (>50%) [21]. We performed subgroup analyses to eliminate the heterogeneity based on the follow up duration. Sensitivity analyses were performed if high heterogeneity was detected. This meta-analysis was performed using Review Manager Software Version 5.3.

The protocol was approved by the human research Ethics Committee, Faculty of Medicine, Prince of Songkla University, Thailand (REC Number: 59-146-18-1).

## Results

### Study selection

**Fig. 1:**
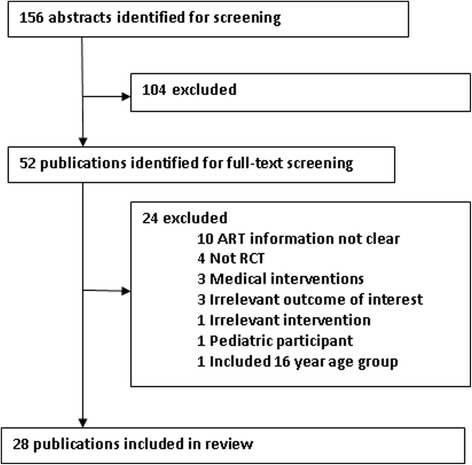
Study selection flowchart

A total of 167 publications were identified from the electronic databases using the search strategy. After excluding duplicate publications, 156 articles remained (Fig. 1). Of these, 104 did not meet the inclusion criteria. Of the 52 remaining publications, 24 were excluded: ten did not report clear information about ART medication, four were not randomized clinical trials (RCTs), three used a medical intervention, three had not quality of life outcome, one had not social or behavioral intervention, one was conducted in a pediatric population, one included participants from 16 years of age, and one included non-HIV participants. Finally, 28 publications were included in this review.

### Study characteristics

Table 4 presents the characteristics of the 28 studies [24–51]. All studies were RCTs and published from 2002 to 2014. Studies were conducted in the following countries: United States of America (15), Canada (1), Switzerland (1), France (1), Spain (1), Brazil (1), Rwanda (1), South Africa (1), Australia (1), China (2), Vietnam (1), Thailand (1), and multicenter settings (1: South Africa, Puerto Rico, USA). Study duration ranged from 6 to 54 months and six studies did not mention the duration of study. The number of participants per study ranged from 22 to 507.

**Table 4:** Characteristics of included trials reporting quality of life of HIV infected people receiving ART

Author, year, location	Study design & setting	Study duration in months	Intervention group	Control group	Intervention facilitator	Group size for intervention	Time per session and total session	Intervention period (wks)	Follow up month	Age mean (SD)	Male	Female	TG	Outcome and tools
Berger et al, 2008 [24], Switzerland	RCT, multicenter, clinic	9	Cognitive behavioral stress management (CBSB)	Standard care	1. Cognitive behavioral psychotherapist 2. Postgraduate psychotherapy trainee	4 to 10	2 h, 12 session	12	1,6,12	44.1 (10.1)	89	15		QoL: MOS-HIV, Anxiety: HADS-A, Depression: HADS-D, CD4, HIV1-RNA, adherence to ART
Blank et al, 2014 [25], USA	Longitudinal randomized trial, community	54	Preventing AIDS through Health for HIV positive persons (PATH+), Psycho-education along with pillboxex and beeping watches	Standard care	Advanced practice nurse	12 to 20/visit	1visit/week	48	3, 6, 12, 24	43.0 (7.25)	128	110		QoL: SF-12, Viral load, CD4
Bormann et al, 2006 [26], USA	RCT, mixed repeated measure, clinic	12	Mantram intervention	Attention group	Psychiatric mental health nurse	8 to 15	1.5 h, 6 session, with phone call 4 time	10	1.1, 2.2, 5.2	42.9 (6.84)	75	18		Qol: Q-LES-Q, Anxiety: STAI, Depression: CES-D, Distress: HIV- IES, Stress: PSS, Anger: STAI-SF, Spiritual well being: FACIT-SpEx
Brown et al, 2014 [27], USA	RCT, clinic (secondary analysis)		System CHANGETM-HIV intervention, self-management, spiritual and optimism	Standard care and symptom management materials	Trained female interventionist	8 to 10	1 h, 10 session	10	2.2	48.4 (6.9)	25	18		Spirituality: SWBS, Optimism: LOTR
Cade et al, 2010 [28], USA	RCT, prospective, clinic		Yoga intervention	Standard care	Certified yoga instructor	Individual and group	1 h, 40–60 session	20	5	45.0 (7.9)	10	50		QoL: MOS-SF-36, Nutritant intake
Chhatre et al, 2013 [29], USA	RCT, single blinded; community	6	Transcendental meditation ™	Healthy eating (HE) education	Certified instructor	Individual and group	2 h, 32 session & 20 min twice a day	24	6	49.9 (5.8)	18	4		QoL: MOS-SF-36, Depression: CESD, Stress: PSS, HIV-QoL: FAHI, Quality of well being: QWB-SA, Hormonal outcome
Duncan et al, 2012 [30], USA	Randomized, wait-list controlled trial; clinic	6	Mindfulness-based stress reduction (MBSR)	Standard care (WLC)	Experienced MBSR teacher	Individual and group	2.5–3 h, 8 session & 1 h home practice	8	3, 6	48.0 (7.9)	64	12		Depression: BDI, Stress: PSS, Positive & negative affects schedule, Mindfulness: FFMQ, ART adherence, Side effect checklist
Eller et al, 2013 [31], South Africa, Puerto Rico, USA	Multisite RCT, clinic & community	13	HIV/AIDS symptom management manual self-care symptom management strategies	Nutritional care and support for PLHIV	Research nurse	Individual	30 min., 1 session		2	43.1 (9.6)	126	93	3	Depressive symptom: CES-D
Fillipas et al, 2006 [32], Australia	RCT, single blinded; clinic and community	27	Supervised aerobic and resistance exercise program	Unsupervised walking program and attended a monthly group forum	Physiotherapist	8 to 10	1 h, 48 session	24	6	43.5 (8.8)	40			QoL: MOS-HIV health survery-35, Self-efficacy: GSES, CD4, Viral load, cardiovascular fitness
Galantino et al, 2005 [33], USA	RCT, clinic	12	Tai Chi (TC) and aerobic exercise (EX)	Standard care	Licensed physical therapist and aerobic instructor	13	1 h, 16 session	8	2		51			QoL: MOS-HIV, Spiritual well being or psychological change: POMS
Gayner et al, 2012 [34], Canada	RCT, clinic	36	Mindfulness-based stress reduction (MBSR)	Standard care	Psychiatrist	Individual and group (14 to 18)	3 h, 8 session, 1 h homework/day	8	2, 6	43.8 (7.0)			117 (Gay men)	Anxiety: HADS, Depression: HADS, Positive & negative affect schedule, mindfullness scale: TMS, Impact of event scale, Distress: IES
Goujard et al, 2003 [35], France	RCT, multicenter, clinic	24	Education	Standard care	Physician and nurse	Individual	1 h, 3 session	48	6, 12, 18	40.5	261	65		QoL: HIV-46, Adherence to ART: PMAQ7, CD4
Jones et al, 2007 [36], USA	2X2 factorial design; clinic & community		Cognitive behavioral stress management (CBSM+) and healthier lifestyle	Group and individual (Factorial groups)	Therapist	Individual and group	2.5 h, 6 session	12	3, 6	41.0 (8.0)		177		Coping with stress: COPE, adherence to ART: ACTG
Lechner et al, 2003 [37], USA	clinic & community		Group based Cognitive behavioral stress management (CBSM+) and expressive supportive therapy	Individual psychoeducational condition	Psychologist	10	2 h, 10 session	10	3	39.7 (7.1)		330		QoL: MOS-HIV-30
Li et al, 2010 [38], Thailand	RCT, clinic	24	Behavioral intervention	Standard care		Group	13 session	13	6, 12	37.4 (6.6)	167	340		QoL: MOS-HIV, Depression, Disclosure, Internalized shame, Social support, Family functioning
Maharaj et al, 2011 [39], South Africa	Randomized controlled prospective longitudinal, clinic	24	Rehabilitation exercise	Heat therapy & reading magazine			1 h, 12 session	12	3	33.6 (9.6)	34	18		QoL: MOS-SF-36
McCain et al, 2003 [40], USA	RCT, community		Cognitive behavioral stress management (CBSM) and social support group (SSG)	Wait list	Mental health nurse	6 to 10	1.5 h, 8 session	8	2, 6	39.4	119	29		QoL: FACT-G, Psychological distress: IES, Perceived stress: DIS, Coping patterns: DIS, Social support: SPS, viral load, CD
Miles et al, 2003 [41], USA	Intervention, clinic	36	HIV self care symptom management	Standard care	Registered nurse	Individual	6 visit	12	1, 6	37.0 (8.4)		109		Qol: MOS-HIV-35, Depression: CESD, Profile mood states: POMS, Stigma: Demi, HIV worry
Molassiotis et al, 2002 [42], China	Intervention, clinic		Cognitive behavioral therapy (CBT) and peer support counseling (PSC)	Standard care	Qualified nurse experienced in counseling	3 to 6	2 h, 12 session	12	3, 6	39.1 (10.8)	32	3		QoL: WHOQOLBRF, Depression, Anger, Uncertainty in illness: MUIS, POMS
Mutimura et al, 2008 [43], Rwanda	RCT, clinic	12	Body fat redistribution (BFR) and exercise training (EXS)	BFR but noexercise training (nEXS)			1.5 h, 72 session	24	6	37.7 (6.2)	40	60		QoL: WHOQOL-HIV, BMI
Ogalha et al, 2011 [44], Brazil	RCT, clinic	6	Physical activities (aerobic, resisted and stretching) and counseling	Counseling	Nutrition specialist	11	1 h, 72 session	24	6	43.0 (9.4)	34	29		QoL: MOS-SF-36, BMI, CD4, Nutritional status
Proeschold-Bell et al, 2010 [45], USA	RCT, clinic	24	Health information exchange	Standard care				98	12, 24	42.4 (7.7)	145	109		QoL: MOS-SF-36, viral load, CD4, adherence to ART
Ruiz et al, 2010 [46], Spain	Randomized, concurrent, follow up study, clinic	12	Treated by health professional (physician or pharmacist with extensive knowledge)	Treated by peer	Trained therapist		1 h, 4 visit	24	2, 4, 6	41.16 (8.16)	176	64		QoL: MOS-HIV-35, Adherence to ART: SMAQ, Psychological distress: GHQ-12, Social support: Duke-UNC-11, Viral load
Sikkema et al, 2005 [47], USA	Community	22	Cognitive behavioral bereavement coping group intervention	Individual psychotherapy upon request	Clinical psychologist, Clinical social workers, Nurse	6 to 8	1.5 h, 12 session	12	3	40.3 (7.0)	150	85		QoL: FACT-G (FAHI), Physical health status
Tam et al, 2012 [48], Vietnam	RCT, cluster; clinic	13	Peer support and standard care	Standard care	Trained HIV infected people	Individual	56 visit	48	12		155	73		QoL: WHOQOL-HIVBREF
Wang et al, 2010 [49], China	RCT; clinic	8	Nursing intervention (home visit and telephone calls)	Standard care	Nurse	Individual	4 session	32	8	36.7 (5.6)	97	19		QoL: WHOQOLBREF, Depression: SDS, Adherence to ART: CPCRA
Webel, 2010 [50], USA	RCT, clinic & community	12	Pee based symptom management and positive self-management program	HIV symptom management strategies	Trained peer leader	10	2 h, 7 session	7	3	47.0 (8.6)		74	14	QoL: HIV/AIDS TQoL, Adherence to ART: ACTG, Symptom intensity
Wu et al, 2006 [51], USA	RCT, clinic	24	Disease management assistance system (DMAS) and education	Adherence counseling		Individual	30 min., 6 session, daily reminder	24	6	38.5 (6.9)	36	26		QoL: MOS-HIV, Depression: CESD, activity of daily living: IADLs, Role functioning: SF-36, Adherence: eDEM, CD4, Viral load

*QoL* quality of life, *TG* transgender

### Intervention characteristics

All 28 studies applied different types of interventions. Control groups for 14 of the studies received standard care (standard or wait-list control or comparison group), and in the remaining studies included group exercise (1), standard routine care and symptom management manual (2), healthy eating education (1), nutritional support and care (1), unsupervised walking program and monthly group forum (1), therapist guided exercise (1), individual psycho-educational condition (1), heat therapy and reading magazine (1), no exercise (1), counseling (1), treatment by peers (1), individual psychotherapy (1), and adherence counseling (1). The studies had a wide variety of intervention sessions and types of interventionists. The intervention period ranged from 7 to 98 weeks and the follow up period ranged from 1 to 24 months. The mean age for all study participants ranged from 33.6 to 49.9 years. Four studies included only females [36, 37, 41, 50], and two included only males [32, 33] (Table 4).

### Study quality or risk of bias

**Fig. 2:**
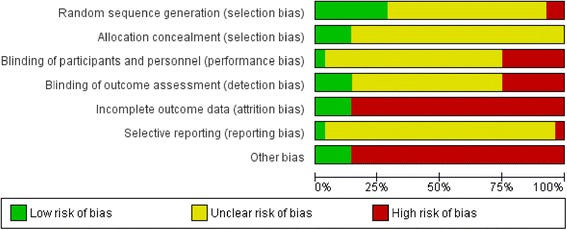
Risk of bias of included studies

Cochrane risk of bias tool was used to determine the quality of methods among all 28 studies (Fig. 2). Eight studies reported details of random sequence generation methods [24, 26, 30, 32–34, 41, 51] and seven studies provided details of their allocation concealment [24, 26, 30, 32, 42, 45, 46]. Ten studies provided information related to participant and personnel or outcome assessment blinding [24–26, 29, 32–34, 37, 39, 46]. Eleven studies did not mention the reasons for participants’ withdrawals [25, 27, 29, 35, 39–41, 43, 44, 50, 51] and 13 studies described an intention-to-treat analysis approach [24, 26, 27, 29, 30, 32–34, 38, 41, 46, 47, 50]. Table 5 shows the key quality of evidence and impact of the individual study.

**Table 5:** Validity, quality and impact of the individual study

ID	Internal validity	External validity	Quality of evidence for individual studies
Berger et al, 2008 [24]	Fair	Fair	Medium
Blank et al, 2014 [25]	Poor	Fair	Medium
Bormann et al, 2006 [26]	Poor	Fair	Medium
Brown et al, 2014 [27]	Fair	Fair	Medium
Cade et al, 2010 [28]	Poor	Poor	Weak
Chhatre et al, 2013 [29]	Fair	Poor	Weak
Duncan et al, 2012 [30]	Fair	Poor	Medium
Eller et al, 2013 [31]	Poor	Poor	Weak
Fillipas et al, 2006 [32]	Fair	Fair	Medium
Galantino et al, 2005 [33]	Poor	Poor	Medium
Gayner et al, 2012 [34]	Fair	Fair	Medium
Goujard et al, 2003 [35]	Poor	Poor	Medium
Jones et al, 2007 [36]	Poor	Poor	Medium
Lechner et al, 2003 [37]	Poor	Poor	Medium
Li et al, 2010 [38]	Fair	Good	Strong
Maharaj et al, 2011 [39]	Poor	Poor	Weak
McCain et al, 2003 [40]	Poor	Poor	Medium
Miles et al, 2003 [41]	Poor	Poor	Medium
Molassiotis et al, 2002 [42]	Poor	Fair	Medium
Mutimura et al, 2008 [43]	Fair	Poor	Medium
Ogalha et al, 2011 [44]	Poor	Poor	Medium
Proeschold-Bell et al, 2010 [45]	Poor	Poor	Medium
Ruiz et al, 2010 [46]	Fair	Fair	Medium
Sikkema et al, 2005 [47]	Fair	Good	Medium
Tam et al, 2012 [48]	Fair	Fair	Medium
Wang et al, 2010 [49]	Poor	Poor	Weak
Webel, 2010 [50]	Fair	Poor	Medium
Wu et al, 2006 [51]	Poor	Fair	Medium

### Quality of life outcome

All studies reported that quality of life was one of the study outcomes. Eight studies mentioned quality of life as the primary outcome [26, 33, 45, 47–51], three studies mentioned quality of life as the secondary outcome [24, 32, 46] and 17 studies did not provide the information that quality of life was measured either primary or secondary outcome. Quality of life was measured using different tools: MOS-HIV health survey (MOS-HIV or MOS-HIV-35 or MOS-HIV-30) [24, 32, 33, 37, 38, 41, 46, 51], SF-36 health survey [28, 29, 39, 44, 45], SF-12 health survey [25], quality of life enjoyment and satisfaction questionnaire (Q-LES-Q) [26], HIV-46 [35], functional assessment of cancer therapy (FACT-G) [40, 47], WHOQOLBREF [42, 49], WHOQOL-HIV [43, 48], HIV/AIDS targeted quality of life instrument [50], positive and negative affects schedule (PANAS) [30, 34], and spiritual well-being scale (SWBS) [27]. Different studies used different instruments to assess quality of life and different instruments cover different dimensions of quality of life. Seven studies reported no intervention effect on any quality of life domain, of which one reported only one domain [26], two reported four domains [31, 35], one reported 10 domains [45], one reported two domains [46], one reported seven domains [50], and one study reported 11 domains [51] in their final results.

**Fig. 3:**
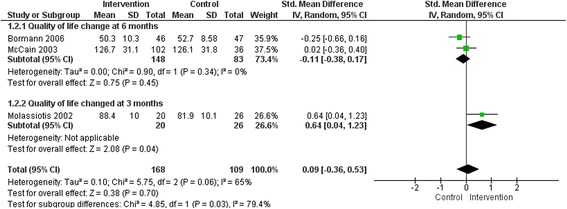
Total quality of life

**Fig. 4:**
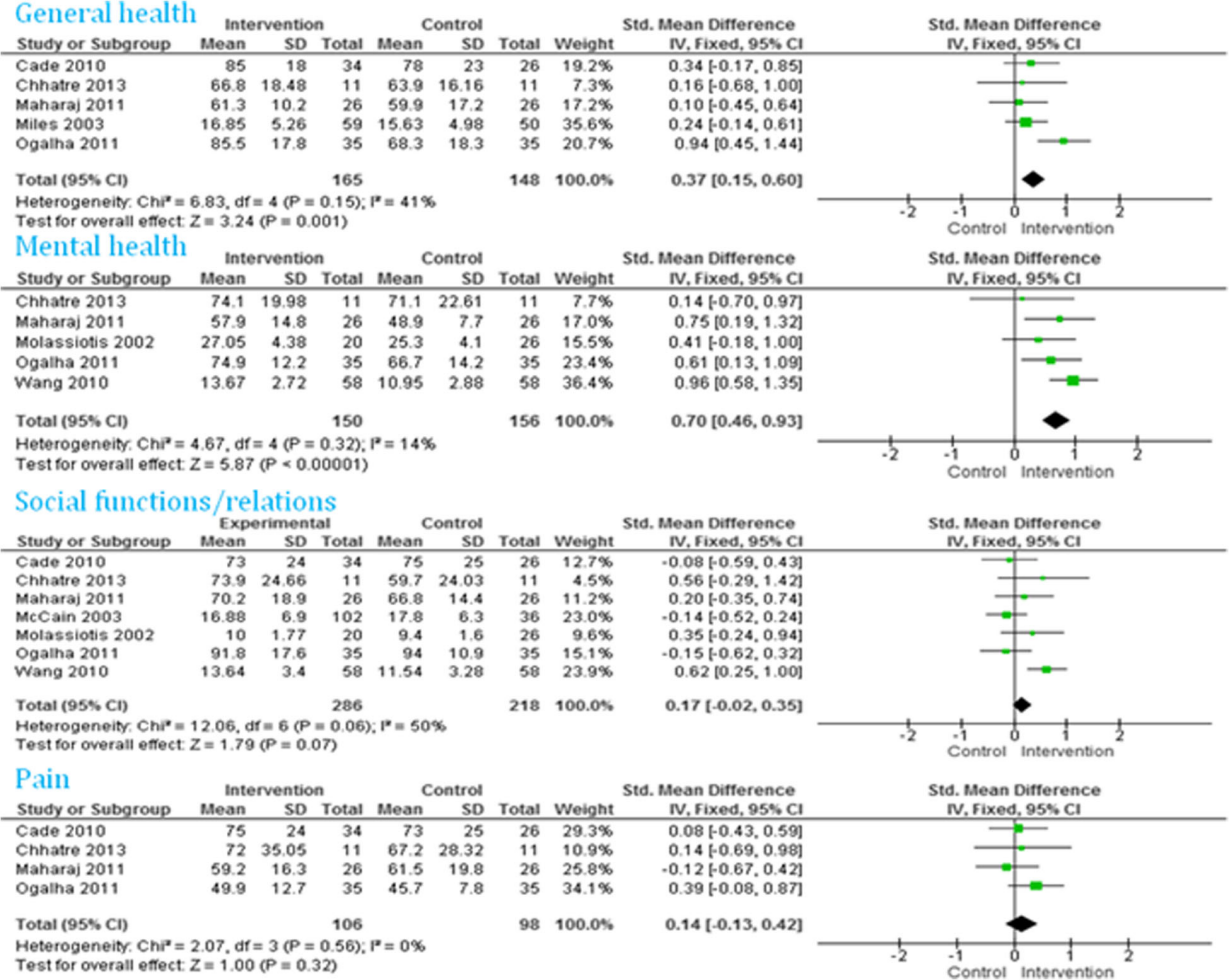
General health, mental health and pain domains of quality of life

**Fig. 5:**
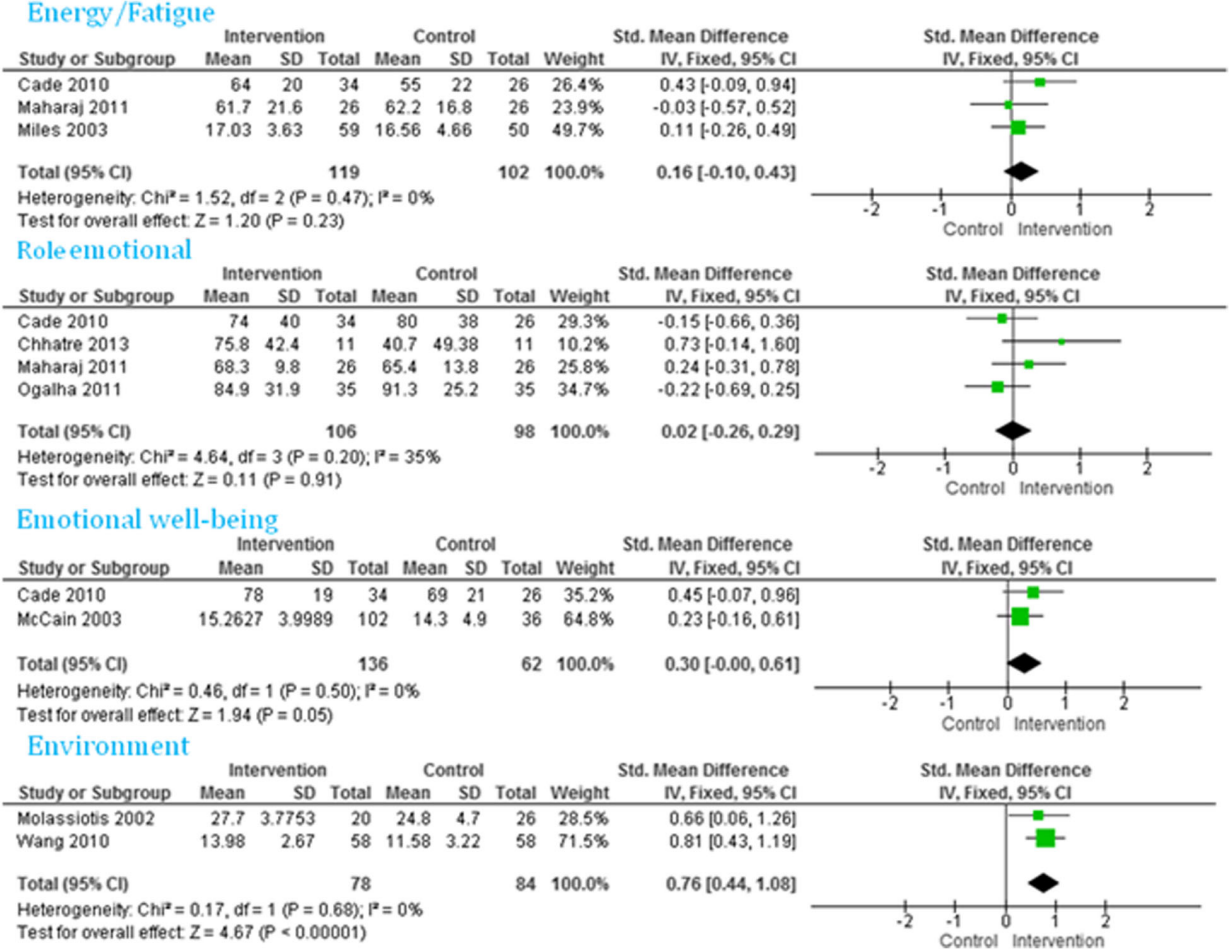
Energy/fatigue, role emotional, emotional well-being and environment domains of quality of life

**Fig. 6:**
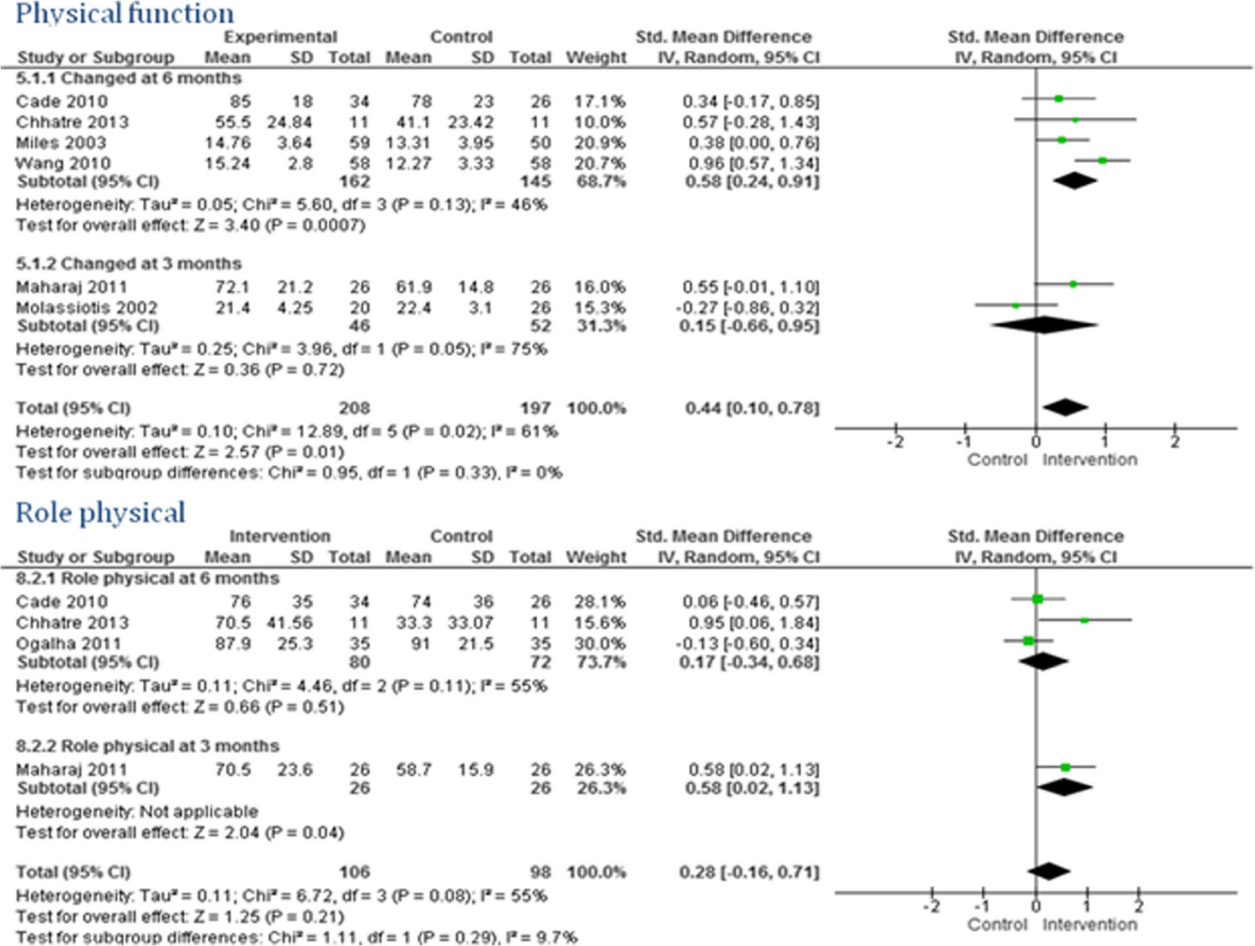
Physical function and role physical domain of quality of life

Twenty-one studies reported a better improvement in quality of life scores among the intervention group compared to the control group. Of these, six studies reported an improvement in all the domains: two out of two domains [24, 25, 27, 40], three out of three domains [48], and four out of four domains [49]. The remaining studies reported that four out of five [41, 43], five out of nine [28], five out of eight [29], seven out of 10 [32], four out of 11 [37], two out of three [38], nine out of 10 [39], two out of four [42], six out of eight [44], and four out of six [47], domains improved among the intervention group. The meta-analyses did not find any overall significant intervention effect in total quality of life (Fig. 3), social function, pain (Fig. 4), energy/fatigue, role emotional, emotional well-being (Fig. 5) and role physical (Fig. 6) domains of quality of life. Significant improvements were found in general health (overall effect: 0.37, 95% confidence interval 0.15, 0.60), mental health (0.70; 0.46, 0.93, Fig. 4), environment (0.76; 0.44, 1.08, Fig. 5) and physical function (0.58; 0.24, 0.91, Fig. 6) domains of quality of life among the intervention group.

### Safety

Only two studies recorded adverse events and no trial reported fidelity of the intervention. A total of 921 participants were lost to follow up or dropped out from the study. Most of the studies did not report the reason for dropping out. Of those, the main reasons were health issues, unavailability of time, emotional issues, no interest, and change of address.

## Discussion

Twenty eight studies included in this review evaluated the impact of social or behavioral interventions on quality of life among HIV infected people with ART. The effects of these interventions should be interpreted with caution since the methodological quality of the studies included in this review was low. Previous systematic reviews highlighted similar findings with methodological issues [18, 19].

In our review, apart from six studies which found improvement in quality of life domains, the expected impact of the intervention was rated as low or moderate based on the available evidence. A previous systematic review highlighted similar findings that evidence was limited to assess the impact of intervention on quality of life [17]. Our findings from the meta-analyses indicated a significant improvement in general health, mental health and physical health domains of quality of life. A previous meta-analysis based on exercise intervention revealed similar findings in that few domains of quality of life were significantly improved by the intervention [20]. Lack of rigorous methods of individual studies and unavailability of sufficient information about recruitment process, blinding, effect size estimation, drop out, and intention to treat, resulted in the expected impact on the outcome being inconclusive.

### Applicability of evidence/programmatic considerations for implementation

Social and behavioral interventions for HIV infected people may improve quality of life along with ART. Intervention effects could be improved through establishment of trustable and sustainable networks with available clinical services and community referral systems. Reliable networking can increase motivational and emotional support to HIV infected people. Implementation of social and behavioral services for HIV infected people is still a challenge.

### Research gaps

There was insufficient evidence to strongly recommend the social and behavioral interventions into clinical practices for improving quality of life among HIV infected people for the following reasons. First, the efficacy of the available social and behavioral interventions is not clear. Therefore rigorous and well-designed studies with large sample sizes to address potential confounding, and long-term follow up are needed. Second, sustainability of the intervention is unclear. Research should address the acceptability, feasibility, applicability and sustainability of interventions. Interventions should be cost-effective and appropriate for local cultures and contexts. Third, most of the studies did not mention validation of quality of life measurement tools. Accuracy of the measurement tools was also unclear. Fourth, evidence is not sufficient for targeting sub-groups of HIV populations. More research is needed to formulate HIV prevention and control strategies and policies. In addition, research in this area is needed to develop empowerment and advocacy that play a role in preventing HIV transmission and treatment discontinuation and unavailability. This may have a direct and indirect impact on quality of life of HIV infected people.

### Limitations of the current review

The ability to draw conclusions regarding the effectiveness of social and behavioral interventions among HIV infected people receiving ART is complex. We searched the studies those were written in English language which could limit the sufficient evidences. The interventions in this review were diverse in terms of target populations, type of interventions, delivery persons, measurement tools, duration of intervention and follow up and study duration. The lack of standard measurement tools limits the capacity to evaluate results from individual studies and make conclusions concerning the strength of the evidence. Furthermore, low sample sizes with low statistical power and lack of rigorous methodologies limit the impact and generalizability of the results. Interventions need to have clear guidelines on delivery, method, place and the persons delivering them. All the interventions should be cost-effective and future research should address this component.

## Conclusions

This review has summarized existing evidence on the effect of social and behavioral interventions in improving the quality of life of HIV infected people receiving ART. Based on our review of available evidence and review criterion, social and behavioral interventions are likely to have a low or moderate impact on quality of life. However the methodological limitations can affect the quality of evidence from included studies. Novel and rigorously designed studies and program monitoring and evaluations on HIV outcomes are needed to evaluate the impact of these interventions on key outcomes for quality of life of HIV infected people.
